# 

**Published:** 1860-03

**Authors:** 


### Receipts for the JOURNAL from January 27, to March 1, 1860.

| | | |
|---|---|---|
| R. Ludlam, Ill., | Vol. 3 | $2 |
| M. M. Eaton, Ill., | " 3 | 2 |
| T. A. Bryan, Ind., | " 3 | 2 |
| J. F. Spain, Ohio, | " 3 | 2 |
| Jno. E. Hanback, Ill., | " 3 | 2 |
| E. C DeForrest, Ill., | " 3 | 2 |
| Hiram Carnahan, Ill., | " 3 | 2 |
| J. B. Corey, Wis., | " 3 | 2 |
| J. N. Denney, Ind., | " 3 | 2 |
| Philip Matthei, Ill., | " 3 | 2 |
| J. B. Baker, Ill., | " 3 | 2 |
| S. S. Buck, Ill., | " 3 | 2 |
| A. A. Pierce, Ill., | " 3 | 2 |
| J. B. Washburn, Ind., | " 3 | 2 |
| Geo. Egbert, Ind., | " 3 | 2 |
| P. S. Richards, Ill., | " 3 | 2 |
| Thos. J. Fritts, Ind., | " 3 | 2 |
| J. B. Felker, Ill., | " 3 | 2 |
| V. S. Thompson, Ill., | " 3 | 2 |
| H. S. Blood, Mass., | " 3 | 2 |
| D. O B. Adams, Ill., | " 3 | 2 |
| Chas. Guibon, Ill., | " 3 | 2 |
| Henry Durham, Wis., | " 3 | 2 |
| H. H. Maynard, Iowa, | " 3 | 2 |
| Thos. P. Seller, Ind., | " 2 | 2 |
| Josiah Robinson, Ind., | " 2 | 2 |
| R. F. Henry, Ill., | " 3 | 2 |
| W. H. Heller, Ill., | " 1–2 | 4 |
| N. Sherman, Ind., | Vol. 3 | $2 |
| G. I. Huey, La., | " 2 | 2 |
| J. H. Wiley, Ill., | " 3 | 2 |
| R. Knohstadt, Ill ½ of | " 2 | 1 |
| M. R. Fowler, Cal., | " 3 | 2½ |
| J. M. Steele, Ill., | " 3 | 2 |
| E. R. Boardman, Ill., | " 3 | 2 |
| C. Brackett, Ind., | " 1–2–3 | 6 |
| Sams & Hamilton, Ill., | " 2 | 2 |
| R. B. Treat, Wis., | " 1–2 | 4 |
| C. M. Robertson, Ill., | " 3 | 2 |
| Thos. Bevan, Ill., | " 2 | 2 |
| Wm. Watson, Wis., | " 2 | 2 |
| Wm. McKnight, Ill. ½ of | " 3 | 1 |
| Robinson & Leachman, Ind | " 3 | 2 |
| E. Y. Nichols, Ill., | " 3 | 2 |
| Calvin West, Ind., | " 3 | 2 |
| G. R. Webster, Ill., | " 3 | 2 |
| R. S. Hallock, Iowa, | " 2 | 2 |
| A. Hager, Ill., | " 3 | 2 |
| Henry A. Youmans, Wis. | " 3 | 2 |
| D. S. Caylor, Ind. | " 2–3 | 4 |
| Wm. R. McMahon, Min. | " 3 | 2 |
| H. D Adams, Wis., | " 3 | 2 |
| Ezekiel Friend, Ill., | " 3 | 2 |
| Hosea Davis, Ill., ½ of | " 3–2 | 3 |
| O. J. Corbiu, Ill., | " 3 | 2 |
| T. D. Fisher, Ill., | " 3 | 2 |

